# The impact of dietary calcium content on phosphorus absorption and retention in growing pigs is enhanced by dietary microbial phytase supplementation

**DOI:** 10.1017/S0007114522001039

**Published:** 2023-03-28

**Authors:** Yixin Hu, Wouter Hendriks, Jurgen van Baal, Jan-Willem Resink, Markus Rodehutscord, Marinus M. Van Krimpen, Paul Bikker

**Affiliations:** 1 Wageningen University & Research, Wageningen Livestock Research, 6700 AH Wageningen, The Netherlands; 2 Wageningen University & Research, Animal Nutrition Group, 6700 AH Wageningen, The Netherlands; 3 Trouw Nutrition Research and Development, Stationsstraat 77, 3811 MH Amersfoort, The Netherlands; 4 Institute of Animal Science, University of Hohenheim, 70599 Stuttgart, Germany

**Keywords:** Calcium, Phosphorus, Phytase, Gastrointestinal tract segments, Pigs

## Abstract

Sixty growing male pigs were used to test the hypothesis that high dietary Ca content reduces P absorption to a greater extent in microbial phytase-supplemented diets via reducing inositol phosphate (IP) degradation and enhancing P precipitation. Pigs were equally allotted over diets with three Ca contents 2·0, 5·8 and 9·6 g/kg with or without microbial phytase (0 *v*. 500 FTU/kg) in a 2 × 3 factorial arrangement. Faeces and urine were collected at the end of the 21-d experimental period. Subsequently, pigs were euthanised and digesta quantitatively collected from different gastrointestinal tract (GIT) segments. Increasing dietary Ca content reduced apparent P digestibility in all GIT segments posterior to the stomach (*P* < 0·001), with greater effect in phytase-supplemented diets in the distal small intestine (*P*_interaction_ = 0·007) and total tract (*P*_interaction_ = 0·023). Nonetheless, increasing dietary Ca to 5·8 g/kg enhanced P retention, but only in phytase-supplemented diets. Ileal IP6 degradation increased with phytase (*P* < 0·001) but decreased with increasing dietary Ca content (*P* = 0·014). Proportion of IP esters in total IP (∑IP) indicated that IP6/∑IP was increased while IP4/∑IP and IP3/∑IP were reduced with increasing dietary Ca content and also with a greater impact in phytase-supplemented diets (*P*_interaction_ = 0·025, 0·018 and 0·009, respectively). In all GIT segments, P solubility was increased with phytase (*P* < 0·001) and tended to be reduced with dietary Ca content (*P* < 0·096). Measurements in GIT segments showed that increasing dietary Ca content reduced apparent P digestibility via reducing IP degradation and enhancing P precipitation, with a greater impact in phytase-supplemented diets due to reduced IP degradation.

P is an essential nutrient for all farmed animals. Most of the P in cereal grains and oil seeds is bound to inositol phosphate (IP)^(1)^ and poorly available for pigs and poultry. Improving gastrointestinal IP breakdown and reducing P excretion have been intensively studied^(2)^ because of finite global mineral P availability, increasing legal pressure on P output and public concerns regarding environmental P pollution including surface water eutrophication. Dietary inclusion of microbial phytase is widely practised to improve gastrointestinal P absorption by intensively farmed, non-ruminant animals.

The impact of dietary Ca content on microbial phytase efficacy has received much attention in earlier studies^(3,4)^ since Lei *et al.*^(5)^ concluded that a normal *v*. a low-Ca level greatly reduced the efficacy of supplemental phytase in pigs. The latter study, however, did not have appropriate control treatments with phytase-free diets to support that conclusion. Based on a meta-analyses of thirty studies in pigs from 1974 to 2009, Létourneau-Montminy *et al.*^(6)^ concluded that the (apparent total tract) digestibility (ATTD) of dietary P decreased with dietary Ca concentration independently of phytase. This study indicates the absence of an interaction between dietary Ca and microbial phytase on ATTD of P. Unfortunately, only few studies made a direct comparison between the effect of dietary Ca level on P digestibility in phytase-free and phytase-supplemented diets. Poulsen *et al.*^(7)^ and Létourneau-Montminy *et al.*^(8)^ showed that ATTD of P was enhanced by microbial phytase, independent of dietary Ca content. In contrast, Brady *et al.*^(9)^ found that microbial phytase supplementation increased ATTD of P in pigs fed low but not high Ca diets, indicating that a high Ca intake reduced microbial phytase efficacy. A similar reduction in phytase efficacy by dietary Ca was observed by Seynaeve *et al.*^(10)^ for total tract but not for precaecal P digestibility. The latter results may indicate that the interaction between dietary Ca and microbial phytase depends on the conditions in the digestive tract and varies between gastrointestinal tract (GIT) segments. Luminal pH gradually increases along the GIT and affects Ca and phytate solubility, phytase activity and their interactions^(4)^. More insight into these interactions and the consequences for Ca and P absorption in different segments of the GIT would contribute to a better understanding of the impact of Ca on phytase efficacy and P absorption and discrepancies between studies as discussed above. However, according to our knowledge, studies addressing these interactions along the GIT have not been published.

We hypothesised that a high dietary Ca content reduces P absorption via reducing IP degradation and enhancing precipitation of P in the small intestine (SI) of pigs, with a greater impact in microbial phytase-supplemented diets. A low-Ca diet can be expected to enhance intestinal P absorption but may reduce P deposition and bone formation. Therefore, the objective of this research was to investigate the interactions between dietary Ca content and microbial phytase supplementation on apparent Ca and P digestibility and solubility in different GIT segments, IP degradation in the distal-SI as well as faecal and urinary Ca and P excretion and retention in growing pigs.

## Materials and methods

The experiment was approved by the ethical committee of Wageningen University & Research (2016.D-0065.006) and conducted in the facilities of the Swine Research Centre of Trouw Nutrition (Sint Anthonis, the Netherlands). All procedures agreed with Dutch laws on animal trials in accordance with EU directive 2010/63. Daily monitoring of the animals was conducted by experienced animal caretakers under supervision of veterinarian.

### Animals, experimental design and diets

Sixty growing male pigs (Hypor Libra × Maxter, 30·4 (sd 1·3) kg) were allocated to a diet containing either 0 or 500 FTU/kg microbial phytase and three levels of dietary Ca (2·0 (low), 5·8 (medium) and 9·6 (high) g/kg) in a 2 × 3 factorial arrangement. The experiment was replicated over time in two periods and consisted of six dietary treatments. In each period, the allocation of pigs was realised by grouping thirty pigs in five replicates with six pigs of similar body weight and randomly allocating one pig per block to each treatment.

Experimental diets (Table 1) were produced by a feed production plant for research diets (ABZ Diervoeding) using a double mixing procedure. Prior to feed production, a mixture of wheat, barley and soyabean meal was heated to 80 °C to deactivate intrinsic phytase activity. Subsequently, a basal diet was made which met or exceeded the minimal requirement of all nutrients except for Ca and P^(11)^. No limestone was added to the basal diet, and monosodium phosphate was used to realise the intended digestible P content (1·7 g/kg, 70% of the requirement for digestible P (2·4 g/kg, CVB^(11)^)). Titanium dioxide (TiO_2_) was added to the basal diet at 4·0 g/kg as an indigestible marker. The basal diet was thoroughly mixed and divided into six equal portions before the required amount of limestone (Sibelco) was added at the expense of diamol (Damolin) and microbial phytase (Axtra Phy; Danisco Animal Nutrition) was added to three diets without reducing another ingredient. Because of the low inclusion level of 0·1 g/kg the diluting effect was negligible. Diet samples were taken automatically during feed production by opening of a valve in the feed processing line after the cooler at regular time intervals and collection of the aliquots of feed in a bucket. From the pooled sample of each diet, three representative subsamples of 500 g each were taken for analysis and storage. The diets were pelleted (4 mm) at a maximum temperature of 80 °C to prevent segregation during shipping and storage. The intended and analysed nutrient contents of the diets are shown in Table 1.

**Table 1.** Ingredient composition and analysed nutrient concentrations of the experimental diets

Phytase addition, FTU/kg	0			500		
Ca content, g/kg as-fed	2·0	5·8	9·6	2·0	5·8	9·6
Ingredients, g/kg as-fed						
Wheat	249	249	249	249	249	249
Barley	249	249	249	249	249	249
Maize	173	173	173	173	173	173
Soyabean meal	117	117	117	117	117	117
Rapeseed meal	80	80	80	80	80	80
Sunflower seed meal	40	40	40	40	40	40
Soyabean oil	32	32	32	32	32	32
Molasses	20	20	20	20	20	20
Premix*	5	5	5	5	5	5
Salt	2	2	2	2	2	2
Amino acids†	6	6	6	6	6	6
Monosodium phosphate	3	3	3	3	3	3
Phytase‡	0	0	0	0·1	0·1	0·1
Limestone§	0	10	20	0	10	20
Diamol||	20	10	0	20	10	0
Titanium dioxide	4	4	4	4	4	4
Calculated composition, g/kg as-fed						
NE, MJ/kg	9·7	9·7	9·7	9·7	9·7	9·7
Crude protein	171	171	171	171	171	171
Crude fat	59	59	59	59	59	59
AID lysine¶,**	9·1	9·1	9·1	9·1	9·1	9·1
Ca	2·0	5·8	9·6	2·0	5·8	9·6
P	4·7	4·7	4·7	4·7	4·7	4·7
IP-P††	2·9	2·9	2·9	2·9	2·9	2·9
Digestible P**	1·7	1·7	1·7	2·5	2·5	2·5
Analysed composition, g/kg as-fed						
DM	889	886	889	888	889	889
Crude ash	57	58	58	57	57	58
Crude protein	181	184	184	184	187	184
Crude fat	58	51	56	58	58	59
Starch	363	364	367	360	374	372
Sugar	46	46	45	46	48	45
Titanium	2·38	2·46	2·33	2·39	2·45	2·32
Ca	1·8	5·3	8·9	1·8	5·3	8·6
P	4·9	4·9	4·9	4·8	4·9	4·8
IP6-P††	2·3	2·3	2·3	2·3	2·3	2·3
IP5-P††	0·2	0·2	0·2	0·2	0·2	0·2
∑IP-P††	2·5	2·5	2·5	2·5	2·5	2·5
Phytase activity, FTU/kg as fed	< 100	< 100	< 100	568	610	547

* Premix contributed per kg of diet: vitamin A 10 000 μg; vitamin D_3_ 2000 μg; vitamin E 40 mg; vitamin K_3_ 1·5 mg; vitamin B_1_ 1·0 mg; vitamin B_2_ 4·0 mg; vitamin B_6_ 1·5 mg; vitamin B_12_ 20 µg; niacin 30 mg; d-pantothenic acid 15 mg; choline chloride 150 mg; folic acid 0·4 mg; biotin 0·05 mg; Fe 100 mg; Cu 20 mg; Mn 30 mg; Zn 70 mg; I 0·70 mg; Se 0·25 mg.

† Provided in g per kg of diet: l-lysine HCl; 4·1; l-threonine; 0·9; dl-methionine; 0·7; l-tryptophan, 0·2.

‡ Axtra Phy, Danisco Animal Nutrition, Marlborough, UK.

§ Sibelco, Maastricht, the Netherlands.

|| Damolin, Kønsborgvej, Denmark.

¶ AID = apparent ileal digestible.

** Calculated according to CVB (2018)^(47)^.

†† ∑IP-P = sum of inositol phosphate (IP) bound P; IP6-P = IP6 bound P; IP5-P = IP5 bound P; inositol tetraphosphate (IP4) and triphosphate (IP3) were not detected in the diets.

### Animal husbandry and feeding strategy

Each of the two experimental periods lasted 21 d and included a 4-d faeces and urine collection period (d 14–18). Pigs were first individually housed in pens containing a slatted floor (d 0–9) and then transferred to individual metabolism pens (d 10–20). During the collection period, faeces were collected in plastic bags connected to the pigs with a Velcro system^(12)^. The bags with faeces were removed from the pigs twice a day in the morning and afternoon and stored at –20 °C. At the end of the collection period, a representative subsample was taken by combining all (i.e. eight) bags with faeces per pig, thoroughly mixing of the faeces, taking a subsample of approximately 500 g, freeze drying and grinding of the subsample and using this material for further analysis. Urine was quantitatively collected in plastic buckets via a funnel mounted underneath the metabolism pens. At the end of balance trial, urine samples were quantitatively pooled per animal, thoroughly mixed, subsampled (2 l per pig) and stored at –20 °C. Environmental temperature (23 (sd 1 °C)) and ventilation were automatically controlled by a climate computer. A dark/light schedule (06.00 to 22.00 hours lights on) was used throughout the entire experimental period, except for the dissection day (d 20) during which time lights were on from 02.00 hour in order to feed the pigs according to a frequent feeding regimen.

Daily feed allowance was three times net energy requirement for maintenance (293 KJ NE/BW^0·75^) based on individual BW measured on d 0, 10 and 20, with daily increments based on the expected body gain. From d 0 to 10, the feeding level was based on the initial BW on d 0 plus an estimated average daily gain of 500 g/d. The overall daily gain from d 0 to 10 was used to estimate the average daily gain from d 10 to 20 and to calculate the feed allowance accordingly. Pigs received two equal meals per day at 07.00 and 15.00 hours from d 0 to 17. From d 18 onwards, pigs were fed according to a frequent feeding regimen adopted from Schop *et al.*^(13)^ and Martens *et al.*^(14)^ to ensure a constant digesta passage rate in the GIT. Briefly, pigs received 1/6^th^ of their daily allowance in six equal meals from 07.00 to 22.00 hours at 3-h intervals on d 18–19. On d 20, feeding frequency was increased to 1-h intervals and pigs received half of their daily allowance equally distributed over six meals until dissection. Pigs were fed and dissected per replicate. Liquid feeding without soaking was used (feed/water 1:2, w/w base) throughout the experiment with the Ca content in water (0·034 g/l) included in the calculation of Ca intake, apparent digestibility and retention. The P content in the water was very low (0·013 mg/l), hence this was not taken into account in the calculations. Additional water was provided in the trough after each meal, with an allowance increasing from 0·3 to 0·7 l to enable the pigs to realise a water intake close to *ad libitum*. Feed refusals were collected, dried and recorded every day.

### Sample collection and chemical analysis

On d 20, pigs were sedated with Zoletil^®^ 100 (0·06 ml/kg BW), weighed and then euthanised via a jugular vein injection of Euthasol^®^ (24 mg/kg BW). Subsequently, the GIT was exposed, carefully taken out and divided into six segments (enclosed by zip ties) including stomach, proximal and distal half of SI, caecum, proximal and distal half of large intestine (LI). The GIT segments were quantitatively emptied by gentle squeezing. After thorough mixing followed by a pH determination (Mettler Toledo), the digesta were immediately stored at −20 °C before further analysis. The caecum and proximal-LI digesta were pooled after pH determination. The lower left thoracic limb was removed at the carpal joint for collection of the 3rd metacarpal bone and stored at −20 °C before analysis.

Diets and faeces were analysed for DM^(15)^, ash^(16)^, crude protein (N^(17)^ × 6·25), crude fat (with acid hydrolysis^(18)^), starch^(19)^ and sugar (all carbohydrates with reducing properties and soluble in 40% ethanol^(20)^). Because of the number of analyses, two methods were used to determine Ca, P and Ti content. A pilot study using six diets and six faeces samples demonstrated non-significant differences between the two methods in the Ca, P and Ti content for both diet and faeces samples irrespective of dietary treatments (data not shown). In faeces, the Ti^(21)^ and P^(22)^ content was determined using a photometer (Thermoscientific) while the Ca^(23)^ content was determined using an atomic absorption spectrometer (Varian). For all other samples (digesta, urine, metacarpal bone ash), the Ca, P and Ti content was determined using the inductively coupled plasma - optical emission spectrometry (ICP-OES, ThermoFisher)^(24)^ after digestion in a microwave (CEM)^(25)^. After thawing in a cooling chamber (4 °C), digesta samples were thoroughly mixed and two representative aliquots were collected. One aliquot was freeze-dried, ground to pass a 1-mm sieve (Retsch GmbH), digested in a microwave (CEM) and analysed for total Ca, P and Ti (ICP-OES)^(24,25)^. The other aliquot was centrifuged at 3000 × ***g*** for 5 min at 4 °C, the supernatant harvested and centrifuged again at 10 000 × ***g*** for 10 min at 4 °C and subsequently analysed for soluble inorganic Ca and P (ICP-OES)^(24)^. No destruction was applied to the supernatant. Because of the high viscosity, distal-LI digesta were diluted with deionised water before centrifugation (1:1, w/w base).

The thoracic limb was thawed, the 3^rd^ metacarpal bone carefully separated using a scalpel, dried in the oven at 70 °C, defatted with petroleum ether, incinerated at 800 °C for ash determination and the ash subsequently destructed in a microwave (CEM) and analysed for Ca and P content (ICP-OES)^(24,25)^.

The IP esters in the diets and distal-SI digesta containing three to six phosphate groups were extracted with 0·2 M EDTA and 0·1 M NaF (pH = 10) before being analysed by high-performance ion chromatography and UV detection at 290 nm with an ICS-3000 System (ThermoFisher) as described by Zeller *et al.*^(26)^. IP1 and IP2 are not determined using this method.

### Calculations and statistical analysis

The apparent Ca and P digestibility coefficient in different GIT segments, IP6 degradation in the distal-SI and ATTD of Ca, P and proximate components were calculated according to de Vries and Gerrits^(27)^:

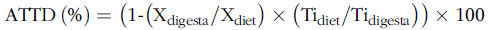




where X_digesta_ and X_diet_ are the analysed content of Ca, P, IP6 and proximate components in the digesta/faeces and diet (g/kg), respectively, with Ti_digesta_ and Ti_diet_ being the analysed Ti content in the digesta/faeces and diet (g/kg), respectively. The Ca content in the diet included the Ca from drinking water based on the water:feed ratio of 2:1.

The retained Ca and P were calculated as:

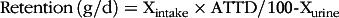




where X_intake_ and X_urine_ are the daily dietary intake and urinary excreted Ca or P (g) during the collection period. The Ca intake included the Ca from water in this calculation based on the water:feed ratio of 2:1.

The Ca and P solubility were calculated as:

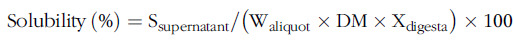




where S_supernatant_ is the soluble inorganic Ca or P content in the supernatant (g), W_aliquot_ is the weight of fresh digesta used for centrifugation (kg), DM is the freeze-dried DM content of the digesta and X_digesta_ is the total Ca or P content in the freeze-dried digesta (g/kg).

The percentage of individual IP esters in the total IP content (∑IP) in the distal ileal digesta was calculated as:

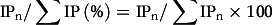




where IP_n_ is the content of individual IP esters (*n =* 3, 4, 5 and 6, mmol/kg) and ∑IP_n_ is the sum of IP_n_ (*n =* 3, 4, 5 and 6, mmol/kg).

Two sample mean power analyses were conducted with the POWER procedure of SAS (version 9.4; SAS Institute), using data from an earlier study to investigate a novel phytase supplementation on apparent Ca and P digestibility in pigs^(28)^. We hypothesised a 3% effect of apparent P digestibility, which required eight replicates to achieve 80 % of power for a two-sided significance level (0·05). Pig was the experimental unit. All data were submitted to a two-way ANOVA using the MIXED procedure of SAS using the following model:

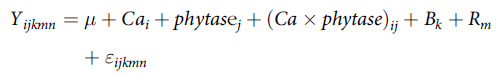




where *Y*_*ijkmn*_ is the dependent variable of the *n*^th^ pig (*n* = 1–60), *µ* is the overall mean, *Ca*_*i*_ is the fixed effect of dietary Ca content (*i* = 1, 2, 3), *phytase*_*j*_ is the fixed effect of dietary phytase level (*j* = 1, 2), (*Ca ×* *phytase*)_*ij*_ is the fixed effect of their interaction, *B*_*k*_ is the random effect of block (*k* = 1–5), *R*_*m*_ is the random effect of period (*m* = 1, 2) and *ϵ*_*ijkmn*_ is the residual error term.

The diagnosis of Studentized residual was visually checked by the graphics plotted using the ODS GRAPHICS function. The LSMEANS procedure with a PDIFF option was used to conduct a post-hoc analysis to separate means of individual treatments in case of a significant interaction between dietary Ca and phytase content and to separate means of the three Ca levels in the absence of this interaction. Probability was considered significant at *P* ≤ 0·05 and a trend at 0·05 < *P* ≤ 0·1.

## Results

All animals remained healthy and completed the study with the exception of one pig (5·8 g/kg Ca with phytase supplementation) in the second period that died of volvulus and one pig (9·6 g/kg Ca without phytase supplementation) in the second period requiring a 1-d medical treatment because of diarrhoea. The analysed Ca, P and Ti content as well as phytase activity in the diets was in good agreement with the targeted levels (Table 1).

### Apparent total tract digestibility and retention of calcium and phosphorus

The P intake of pigs was similar for all treatment groups (Table 2). Overall, increasing dietary Ca reduced intestinal P absorption and urinary P excretion, with a greater impact in the phytase-supplemented compared with phytase-free diets as indicated by the interaction (*P*_interaction_ = 0·023 and *P*_interaction_ < 0·001, respectively). Increasing dietary Ca content from 2·0 to 9·6 g/kg reduced ATTD of P by 6 *v*. 15% units in the phytase-free and phytase-supplemented diets, respectively, with ATTD P values of the phytase-free diets being approximately half of the corresponding values of the phytase-supplemented diets. As a result, P retention was also affected by the interaction between dietary Ca content and phytase inclusion (*P*_interaction_ = 0·002 and *P*_interaction_ < 0·001 for retained P (g/d) and retained P/digested P (%), respectively). Specifically increasing dietary Ca content from 2·0 to 5·8 g/kg increased P retention in the phytase-supplemented but not the phytase-free diets. Overall, phytase inclusion consistently enhanced the ATTD and retention of P with a magnitude depending on the dietary Ca content.

**Table 2.** Mean intake, faecal and urinary excretion, retention and apparent total tract digestibility (ATTD) of P of growing pigs as affected by dietary Ca content and microbial phytase supplementation*,†,‡

Ca content g/kg	Phytase content FTU/kg	P intake g/d	Faecal P g/d	Urinary P g/d	rP g/d	rP/dP %	rP/P intake %	ATTD %
2·0	0	6·9	4·9^b^	0·32^b^	1·7^d^	84·2^c^	25·0^cd^	29·7^d^
5·8	0	6·9	5·0^ab^	0·04^c^	1·8^d^	98·0^a^	26·4^c^	27·0^d^
9·6	0	6·8	5·2^a^	0·02^c^	1·6^d^	98·7^a^	23·2^d^	23·4^e^
2·0	500	6·9	2·7^e^	1·41^a^	2·7^c^	65·5^d^	39·5^b^	60·1^a^
5·8	500	7·0	3·3^d^	0·22^b^	3·4^a^	93·9^b^	48·8^a^	52·0^b^
9·6	500	6·9	3·7^c^	0·02^c^	3·1^b^	99·4^a^	45·5^a^	45·8^c^
sem		0·06	0·10	0·066	0·12	1·82	1·60	1·37
*P*								
Ca content		0·116	< 0·001	< 0·001	< 0·001	< 0·001	< 0·001	< 0·001
Phytase content		0·570	< 0·001	< 0·001	< 0·001	< 0·001	< 0·001	< 0·001
Ca content × phytase content		0·258	0·006	< 0·001	0·002	< 0·001	0·001	0·023

^a^^–^^e^Values without common superscript within a column differ significantly (*P* ≤ 0·05).

* Dietary P content was fixed at 4·7 g/kg of diet; hence, the Ca/P ratio was 0·4, 1·2 and 2·0 for the three increasing dietary Ca contents.

† rP = retained P (g/d); dP = apparently digested P (g/d).

‡ Values are presented as least square means and pooled standard errors of the mean, *n* 10.

The Ca intake increased (*P* < 0·001) with increasing dietary Ca content and was not affected (*P* = 0·290) by phytase supplementation of the diets (Table 3). Phytase increased ATTD of Ca with a greater effect in diets with a lower Ca content (*P*_interaction_ < 0·001). Increasing dietary Ca content increased and reduced ATTD of Ca in the phytase-free and phytase-supplemented diets, respectively (*P*_interaction_ < 0·001). Both faecal and urinary Ca excretion were reduced by phytase inclusion and increased with increasing dietary Ca content (*P* < 0·001). The impact of increasing dietary Ca content on urinary Ca excretion was greater for the phytase-free compared with phytase-supplemented diets (*P*_interaction_ < 0·001). Increasing dietary Ca content increased retained Ca (g/d) but reduced retained Ca as percentage of digestible (retained Ca/dCa) or total (retained Ca/Ca) Ca intake. The magnitude of these effects depended on the phytase supplementation of the diets (*P*_interaction_ < 0·001).

**Table 3.** Mean Ca intake, faecal and urinary excretion, retention and apparent total tract digestibility (ATTD) of growing pigs as affected by dietary Ca content and microbial phytase supplementation*,†,‡

Ca content g/kg	Phytase content FTU/kg	Ca intake g/d	Faecal Ca g/d	Urinary Ca g/d	rCa g/d	rCa/dCa %	rCa/Ca intake %	ATTD %
2·0	0	2·6	1·6	0·08^d^	0·9^d^	91·0^b^	33·7^d^	36·9^e^
5·8	0	7·6	4·4	1·17^c^	2·0^c^	63·2^d^	26·6^e^	42·0^d^
9·6	0	12·4	7·4	2·59^a^	2·4^b^	48·3^e^	19·6^f^	40·6^de^
2·0	500	2·6	0·7	0·05^d^	1·8^c^	97·4^a^	69·5^a^	71·3^a^
5·8	500	7·6	3·0	0·10^d^	4·5^a^	97·8^a^	58·8^b^	60·1^b^
9·6	500	12·5	6·2	1·60^b^	4·7^a^	74·2^c^	37·4^c^	50·2^c^
sem		0·08	0·17	0·132	0·18	3·11	2·01	1·85
*P*								
Ca content		< 0·001	< 0·001	< 0·001	< 0·001	< 0·001	< 0·001	< 0·001
Phytase content		0·290	< 0·001	< 0·001	< 0·001	< 0·001	< 0·001	< 0·001
Ca content × phytase content		0·474	0·227	< 0·001	< 0·001	< 0·001	< 0·001	< 0·001

^a–f^Values without common superscript within a column differ significantly (*P* ≤ 0·05).

* Dietary P content was fixed at 4·7 g/kg of diet; hence, the Ca/P ratio was 0·4, 1·2 and 2·0 for the three increasing dietary Ca contents.

† rCa = retained Ca (g/d); dCa = apparently digested Ca (g/d).

‡ Values are presented as least square means and pooled standard errors of the mean, *n* 10.

### Apparent calcium and phosphorus digestibility in different gastrointestinal tract segments

The apparent P digestibility was improved by phytase inclusion in all GIT segments investigated and was reduced by increasing dietary Ca content posterior to the stomach (Table 4). A significant interaction between phytase and dietary Ca content on apparent P digestibility was observed in the distal ileum (*P*_interaction_ = 0·007), where a dietary Ca content of 9·6 compared with 2·0 g/kg reduced apparent P digestibility by 5 *v*. 15% units in the phytase-free and phytase-supplemented diets, respectively. Apparent digestibility of P was low in the stomach, gradually increased along the SI followed by a reduction in the proximal-LI, being on average 6·4, 21·3, 38·9, 26·2 and 38·9% in the stomach, proximal-SI, distal-SI, proximal-LI and distal-LI, respectively. The apparent P digestibility was much higher in the distal compared with the proximal-LI, particularly for the phytase-free diets.

**Table 4.** Dietary P and Ca apparent digestibility (%) in difference gastrointestinal tract segments in growing pigs as affected by dietary Ca content and microbial phytase supplementation*,†

Ca content g/kg	Phytase content FTU/kg	P					Ca				
		Stomach	Small intestine		Large intestine		Stomach	Small intestine		Large intestine		
			Proximal	Distal	Proximal	Distal		Proximal	Distal	Proximal	Distal
2·0	0	1·0	13·6	25·8^c^	16·4	28·6	–15·9	23·9^c^	11·9^d^	24·6^c^	29·5^c^
5·8	0	5·2	11·8	25·0^c^	0·68	18·5	–2·9	34·8^b^	28·9^c^	23·1^c^	37·5^c^
9·6	0	3·8	–0·7	20·9^c^	3·22	18·0	0·7	36·1^b^	28·8^c^	44·6^b^	53·6^b^
2·0	500	10·7	43·4	62·9^a^	56·2	66·2	–14·2	60·1^a^	64·3^a^	62·3^a^	72·9^a^
5·8	500	13·4	36·1	50·9^b^	46·7	55·5	3·9	51·4^a^	55·9^b^	54·2^ab^	62·4^b^
9·6	500	5·1	23·9	47·7^b^	34·5	47·0	0·6	40·1^b^	43·7^b^	40·9^b^	55·9^b^
sem		3·78	5·37	2·65	4·74	3·31	5·51	5·27	5·06	6·41	4·59
*P*											
Ca content		0·232	< 0·001	< 0·001	< 0·001	< 0·001	< 0·001	0·367	0·428	0·463	0·210
Phytase content		0·007	< 0·001	< 0·001	< 0·001	< 0·001	0·384	< 0·001	< 0·001	< 0·001	< 0·001
Ca content × phytase content		0·273	0·717	0·007	0·137	0·157	0·649	< 0·001	< 0·001	< 0·001	< 0·001

^a–d^Values without common superscript within a column differ significantly (*P* ≤ 0·05).

* Dietary P content was fixed at 4·7 g/kg of diet; hence, the Ca/P ratio was 0·4, 1·2 and 2·0 for the three increasing dietary Ca contents.

† Values are presented as least square means and pooled standard errors of the mean, *n* 10.

In all GIT segments posterior to the stomach, a significant interaction was observed between dietary Ca content and microbial phytase supplementation on apparent Ca digestibility. Increasing dietary Ca content increased apparent Ca digestibility posterior to the stomach in phytase-free diets but reduced it in phytase-supplemented diets (*P*_interaction_ < 0·001, Table 4). Phytase inclusion increased apparent Ca digestibility (*P* < 0·001) in all GIT segments posterior to the stomach. The apparent Ca digestibility was negative in the stomach for the diets with the lowest Ca content (–15·1%) and substantially increased in the proximal-SI (on average 41·0%) followed by a reduction in the distal-SI for the phytase-free but not the phytase-supplemented diets (23·2 and 54·6% for the phytase-free and phytase-supplemented diet, respectively). Moreover, apparent digestibility of Ca was much higher in the distal-LI compared with the distal-SI (on average 52·0 and 38·9%, respectively), especially for the phytase-free diets.

### Calcium and phosphorus solubility

Overall, the solubility of inorganic P gradually decreased along the GIT segments, with a sharp reduction in the distal compared with proximal-SI, being on average 49·8, 49·2, 24·8, 19·7 and 21·7% in the stomach, proximal-SI, distal-SI, proximal-LI and distal-LI, respectively (Table 5). No interaction between dietary Ca content and phytase inclusion on P solubility was observed for any of the GIT segments (*P*_interaction_ > 0·10). In all GIT segments, increasing dietary Ca content reduced (*P* < 0·01) or tended (*P* < 0·10) to reduce P solubility while phytase (*P* < 0·001) increased P solubility.

**Table 5.** Mean solubility (%) of inorganic P and Ca in digesta of difference gastrointestinal tract segments in growing pigs as affected by dietary Ca content and microbial phytase supplementation*,†

Ca content g/kg	Phytase content FTU/kg	P					Ca				
		Stomach	Small intestine		Large intestine		Stomach	Small intestine		Large intestine	
			Proximal	Distal	Proximal	Distal		Proximal	Distal	Proximal	Distal
2·0	0	43·7	56·8	15·0	20·2	21·7	43·6	43·8	3·8	9·3^a^	5·4
5·8	0	47·3	28·9	12·2	15·6	15·9	49·9	21·6	5·8	4·4^b^	2·7
9·6	0	39·5	32·4	14·6	14·0	16·1	48·7	41·2	9·1	4·8^b^	4·2
2·0	500	55·4	70·8	44·0	26·3	29·0	43·1	30·2	6·8	6·9^b^	5·3
5·8	500	57·8	51·3	31·8	24·0	28·7	56·1	32·7	9·2	9·4^a^	5·2
9·6	500	55·1	54·5	30·6	18·1	19·1	54·4	50·0	11·1	5·7^b^	5·2
sem		2·94	7·15	5·18	1·72	3·39	3·42	10·69	2·62	1·12	1·09
*P*											
Ca content		0·052	0·001	0·096	< 0·001	0·009	< 0·001	0·095	0·045	0·003	0·219
Phytase content		< 0·001	0·001	< 0·001	< 0·001	< 0·001	0·057	0·633	0·072	0·089	0·082
Ca content × phytase content		0·454	0·631	0·206	0·244	0·149	0·304	0·170	0·936	< 0·001	0·289

^a,b^Values without common superscript within a column differ significantly (*P* ≤ 0·05).

* Dietary P content was fixed at 4·7 g/kg of diet; hence, the Ca/P ratio was 0·4, 1·2 and 2·0 for the three increasing dietary Ca contents.

† Values are presented as least square means and pooled standard errors of the mean, *n* 10.

The Ca solubility was high in the stomach, lower in the proximal-SI and decreased sharply in the distal-SI, being on average 49·2, 36·6 and 7·6% in stomach, proximal-SI and distal-SI, respectively (Table 5). Increasing dietary Ca content (*P* < 0·05) increased Ca solubility in the stomach and distal-SI, with less consistent effects in the other segments. Phytase (*P* < 0·10) tended to increase the Ca solubility in the stomach, distal-SI and distal-LI with less consistent effects in the other GIT segments.

### Inositol phosphate esters in the distal-small intestine digesta

Microbial phytase supplementation significantly increased dietary IP6 degradation from an average of 5·8–78·6% and reduced ∑IP content in the freeze-dried digesta of the distal-SI (*P* < 0·001; Table 6). The IP6 was largely degraded in the phytase-supplemented diets, with an average proportion of individual IP in ∑IP of 25·8, 7·2, 37·3 and 29·7% for IP6, IP5, IP4 and IP3, respectively. In contrast, in the phytase-free diets, IP6 was largely intact and represented 76·5% of ∑IP. Dietary Ca addition significantly reduced IP6 degradation but not ∑IP in the digesta (*P* = 0·014 and 0·137, respectively). Moreover, increasing dietary Ca content from 2·0 to 9·6 g/kg increased IP6/∑IP to a greater extent in the phytase-supplemented compared with phytase-free diets (16 *v*. 4% units, *P*_interaction_ = 0·025). Increasing dietary Ca content also reduced IP4/∑IP and IP3/∑IP but only for the phytase-supplemented diets (*P*_interaction_ = 0·018 and 0·009, respectively). The IP5/∑IP was inconsistently affected by dietary Ca content (*P*_interaction_ = 0·001).

**Table 6.** Mean inositol phosphate (IP) degradation, total IP content (∑IP) and percentage of different IP esters in freeze-dried distal small intestinal digesta of growing pigs as affected by dietary Ca content and microbial phytase supplementation*,†

Ca content g/kg	Phytase content FTU/kg	IP6 degradation %	∑IP content mmol/kg	IP3/∑IP %	IP4/∑IP %	IP5/∑IP %	IP6/∑IP %
2·0	0	8·4	46·3	2·6^c^	6·2^c^	18·2^a^	72·9^b^
5·8	0	3·0	45·0	1·0^c^	4·2^c^	15·5^b^	79·3^a^
9·6	0	6·1	41·2	1·0^c^	5·5^c^	16·4^ab^	77·2^ab^
2·0	500	88·9	26·7	37·5^a^	39·4^a^	5·8^d^	17·4^e^
5·8	500	73·9	36·1	24·0^b^	39·4^a^	9·6^c^	26·9^d^
9·6	500	73·1	28·6	26·9^b^	33·5^b^	6·4^d^	33·3^c^
sem		5·08	3·89	2·71	1·82	1·19	2·99
*P*							
Ca content		0·014	0·137	0·001	0·033	0·387	< 0·001
Phytase content		< 0·001	< 0·001	< 0·001	< 0·001	< 0·001	< 0·001
Ca content × phytase content		0·168	0·170	0·009	0·018	0·001	0·025

^a–e^Values without common superscript within a column differ significantly (*P* ≤ 0·05).

* Dietary P content was fixed at 4·7 g/kg of diet; hence, the Ca/P ratio was 0·4, 1·2 and 2·0 for the three increasing dietary Ca contents.

† Values are presented as least square means and pooled standard errors of the mean, *n* 10.

### Composition of the 3^rd^ metacarpal bone

The defatted mass of the 3^rd^ metacarpal bone was increased by both dietary Ca content and phytase (*P* = 0·001, Table 7). A significant interaction between dietary Ca content and phytase supplementation was observed on ash content (*P*_interaction_ = 0·034). Increasing dietary Ca content from 2·0 to 5·8 g/kg increased ash content in phytase-supplemented but not in phytase-free diets; further increasing dietary Ca content from 5·8 to 9·6 g/kg resulted in a reduced ash content in the phytase-free but not in phytase-supplemented diets. In addition, the metacarpal Ca mass was enhanced with increasing dietary Ca content irrespective of dietary phytase addition (*P* < 0·001), whereas the P mass was enhanced with dietary Ca only in phytase-supplemented diets (*P*_interaction_ = 0·034). The Ca/P in the metacarpal bone ash was not significantly affected by dietary phytase and Ca content, although a tendency was observed (*P* = 0·056) for an increase with increasing dietary Ca content.

**Table 7.** Mean defatted mass, ash content in fat-free DM, Ca and P mass and Ca/P in ash of the 3^rd^ metacarpal bones in growing pigs as affected by dietary Ca content and microbial phytase supplementation*,†

Ca content g/kg	Phytase content FTU/kg	Defatted mass g	Ash content %	Ca mass g	P mass g	Ca/P in ash
2·0	0	3·4	56·9^cd^	0·378	0·183^b^	2·06
5·8	0	3·6	57·5^bc^	0·405	0·190^b^	2·13
9·6	0	3·7	56·7^d^	0·434	0·195^b^	2·27
2·0	500	3·6	57·0^cd^	0·408	0·194^b^	2·10
5·8	500	4·2	58·5^a^	0·492	0·240^a^	2·06
9·6	500	4·0	58·1^ab^	0·485	0·228^a^	2·12
sem		0·15	0·03	0·021	0·009	0·07
*P*						
Ca content		0·001	< 0·001	< 0·001	0·001	0·056
Phytase content		< 0·001	< 0·001	< 0·001	< 0·001	0·173
Ca content × phytase content		0·329	0·034	0·219	0·034	0·189

^a–d^Values without common superscript within a column differ significantly (*P* ≤ 0·05).

* Dietary P content was fixed at 4·7 g/kg of diet; hence, the Ca/P ratio was 0·4, 1·2 and 2·0 for the three increasing dietary Ca contents.

† Values are presented as least square means and pooled standard errors of the mean, *n* 10.

### Growth performance and apparent total tract digestibility of proximate components

Dietary Ca content reduced (*P* = 0·016) whereas phytase increased (*P* = 0·025) average daily gain from d 0 to 21 (Table 8). Average daily feed intake was not affected by dietary Ca or phytase. The gain to feed ratio was increased by an increasing dietary Ca content (*P* = 0·004) and reduced by dietary phytase addition (*P* = 0·002). Dietary Ca supplementation reduced the ATTD of crude fat (*P* < 0·001; Table 8). The ATTD of ash was increased by both dietary Ca and phytase inclusion (*P* < 0·001), while the ATTD of crude protein and organic matter was not affected by the dietary treatments.

**Table 8.** Mean growth performance parameters from d 0 to 20 of growing pigs and apparent total tract digestibility (ATTD) of proximate components as affected by dietary Ca content and microbial phytase supplementation*,†

Ca content g/kg	Phytase content FTU/kg	Growth performance			ATTD of proximate components, %				
		ADG g/d	ADFI kg/d	G/F g/g	DM	CP	Ash	OM	CFat
2·0	0	736	1·29	0·57	81·1	79·3	22·6^e^	85·1	81·6
5·8	0	700	1·30	0·54	82·2	80·8	32·5^d^	85·6	81·2
9·6	0	642	1·27	0·50	81·9	80·9	38·2^c^	85·0	78·3
2·0	500	729	1·27	0·57	82·0	78·9	33·5^d^	85·3	81·4
5·8	500	752	1·31	0·57	82·8	81·1	39·9^b^	85·7	80·8
9·6	500	712	1·28	0·56	82·3	80·6	45·9^a^	84·8	78·7
sem		28·1	0·023	0·016	0·47	1·32	0·74	0·50	0·97
*P*									
Ca content		0·016	0·208	0·004	0·026	0·123	< 0·001	0·104	< 0·001
Phytase content		0·025	0·742	0·002	0·034	0·834	< 0·001	0·891	0·852
Ca content × phytase content		0·132	0·463	0·103	0·687	0·932	0·002	0·841	0·800

ADFI, average daily feed intake; ADG, average daily gain; CFat, crude fat; CP, crude protein; F/G, feed to gain ratio; OM, organic matter.

^a–e^Values without common superscript within a column differ significantly (*P* ≤ 0·05).

* Dietary P content was fixed at 4·7 g/kg of diet; hence, the Ca/P ratio was 0·4, 1·2 and 2·0 for the three increasing dietary Ca contents.

† Values are presented as least square means and pooled standard errors of the mean, *n* 10.

## Discussion

The hypothesis that a high dietary Ca content reduces P absorption to a greater extent in the phytase-supplemented diets via reducing IP degradation and enhancing precipitation of P in pigs was partly supported by the results of this study. Increasing dietary Ca content from 2·0 to 9·6 g/kg reduced ATTD of P by 6 *v*. 15% units in the phytase-free and phytase-supplemented diets, respectively (Table 2). This phytase-dependent reduction in apparent P digestibility with increasing dietary Ca content was present from the distal-SI (Table 4), confirming the hypothesis of the study. The profile of IP esters in the distal-SI further indicated that increasing dietary Ca content reduced apparent P digestibility via reducing IP6 degradation, with a greater impact on IP6/∑IP in the phytase-supplemented compared with phytase-free diets (16 *v*. 4% units, Table 6). In all GIT segments, increasing dietary Ca content reduced P solubility independent of microbial phytase supplementation (Table 5). It can be concluded that a high dietary Ca content reduced apparent P digestibility via reduction of IP degradation and increased precipitation of P, with a greater impact on IP degradation for the phytase-supplemented diets.

### Calcium and phytase interaction

A high dietary Ca content might inhibit phytase efficacy via Ca-IP complexation and pH buffering. Both Ca-IP complexation and phytase efficacy are pH dependent. Digesta pH gradually increased along the GIT with a mean pH of 3·6, 5·9 and 6·4 observed in the stomach, proximal-SI and distal-SI, respectively (online Supplementary Table S1). Complexation of Ca-IP at a pH below 5 is negligible^(3)^; hence, Ca-IP complexation can be expected to predominantly have occurred in the SI but not in the stomach. In addition, digesta pH was increased with increasing dietary Ca content in the stomach, caecum and proximal-LI (online Supplementary Table S1), while IP is better soluble at a low compared with a high pH^(29)^. The phytase used in the present study was reported by the manufacturer to have the highest efficacy at a pH of 3·5–4·5 and a gradual decrease in efficacy below and above this range. Thus, the limestone used to increase dietary Ca content, with its high pH buffering capacity, might also inhibit phytase efficacy by increasing digesta pH and reducing IP solubility.

Previous studies in broilers indicated that a high compared with low dietary Ca content reduced ileal IP degradation accompanied with a reduced mucosal phosphatase activity^(30)^. Impact of dietary Ca content on mucosal phosphatases expression in pigs has not been reported before. To clarify this aspect, we measured the mRNA expression of two endogenous mucosal phosphatases using RT-qPCR technology. The candidate genes were multiple inositol polyphosphate phosphatase 1^(31)^ and intestinal alkaline phosphatase^(32)^. The expression of both phosphatases was not affected by dietary Ca content or phytase supplementation in the jejunal and colonic mucosa (Hu *et al.* unpublished). A literature survey indicated that precaecal IP6 degradation upon feeding diets devoid of phytase is remarkably lower in pigs than in broilers, suggesting that endogenous phosphatases can be more active in broilers than pigs^(33)^. This might explain why effects of Ca supplementation were observed in the aforementioned broiler study^(30)^ but not in the present study. Brun *et al.*^(34)^ reported that luminal Ca binds and aggregates intestinal alkaline phosphatase but has no impact on the expression level of intestinal alkaline phosphatase in the SI of rats; the present study in pigs agrees with these results. As such a high dietary Ca content does not have an impact on the expression level of mucosal phosphatases but might inhibit their activity to hydrolyse IP in the GIT of pigs.

Results of the present study indicate that a high Ca intake reduced the efficacy of microbial phytase to improve P absorption, conflicted with Poulsen *et al.*^(7)^ and Létourneau-Montminy *et al.*^(8)^. Dietary Ca content was 4 *v*. 8 g/kg and 7 *v*. 10 g/kg in Poulsen *et al.*^(7)^ and Létourneau-Montminy *et al.*^(8)^, respectively. Hence, the contrast of dietary Ca in these two studies was not as great as that in the present study (2·0 *v*. 9·6 g/kg). Besides, Poulsen *et al.*^(7)^ used wheat and barley to formulate their basal diet, which resulted in a high intrinsic phytase activity (650 FTU/kg) that might have masked the microbial phytase efficacy (750 FTU/kg). Wheat and barley were also used in the present study, but these ingredients were preheated at 80 °C before diet inclusion to minimise the intrinsic phytase activity, as confirmed by the analysed phytase activity below the detection limit of the assay in the diets not supplemented with phytase (Table 1). Létourneau-Montminy *et al.*^(8)^ used a basal diet containing a higher digestible P content (2·6 g/kg) than our study, which might partly explain the difference between Létourneau-Montminy *et al.*^(8)^ and the present study. As the impact of dietary Ca content on microbial phytase activity is dependent on diet type, our conclusion that a high dietary Ca content reduces microbial phytase efficacy is valid in the condition of a low intrinsic phytase activity and dietary P content being marginally deficient.

Formation of an insoluble complex of Ca and inorganic P might be another mechanism by which dietary Ca can reduce phytase efficacy. However, this was not supported by our results, because on average the P disappearance represented 94% of the IP-P disappearance in the distal-SI in the presence of microbial phytase in this study. Thus, the released IP-P appeared to have been readily and effectively absorbed in the GIT of the pigs. Phytase supplementation remarkably increased IP6 disappearance, while its impact on apparent P digestibility was smaller. However, phytase supplementation did not completely dephosphorylate the inositol ring as shown by the enrichment of IP3 and IP4 in the presence of phytase compared with the diets without added phytase. Phosphate bound in these IP esters, probably extended by IP2 and IP1 which were not analysed herein, remained undigested and caused the responses in apparent P digestibility to be smaller than IP6 degradation. This is consistent with results by Rosenfelder-Kuon *et al.*^(35)^ and Lu *et al.*^(36)^ who reported an increased concentration of IP3 and IP4 in the ileal digesta of pigs when phytase was supplemented to the feed, even at activities (3000 FTU/kg) that were higher than in the present study.

An interaction between dietary Ca content and microbial phytase was also observed for apparent Ca digestibility (Table 3). This can be explained by the effects of endogenous Ca on ATTD values as shown by González-Vega *et al.*^(37)^. Dietary Ca derived from cereal grains and oil seed meal in the basal diet presumably was chelated with IP causing a low apparent Ca digestibility (36·9%) in pigs fed the low-Ca basal diet (no limestone added) without microbial phytase. The ATTD of Ca only increased by 5·1 and 3·7% as a result of Ca addition of 3·8 and 7·6 g/kg DM by limestone, respectively. While increasing the dietary Ca content, it reduced the influence of basal endogenous losses of Ca, thus marginally affecting the ATTD of Ca. Microbial phytase supplementation hydrolysed dietary IP and significantly increased ATTD of Ca in the low-Ca basal diet (36·9 *v*. 71·3%). Supplementation of Ca from limestone reduced the ATTD of total dietary Ca in phytase-supplemented diets because of the lower digestibility of Ca from limestone and the overall reduction in Ca digestion with increasing dietary Ca.

### Calcium and phosphorus absorption along the gastrointestinal tract

Ca was primarily absorbed in the proximal-SI and LI. Recently, the stomach was suggested to be responsible for approximately 30% of the apparent total tract Ca absorption ^(38; 39)^. In contrast, our results showed that gastric apparent Ca absorption was minor. The discrepancy may be due to surgical preparation of the pigs with T-cannulas in the duodenum and distal ileum in the latter two studies but not in the present study. Cannulation might have affected GIT motility and digesta mean retention in their studies, hence resulting in a greater Ca absorption in the stomach comparing to pigs under normal physiological conditions. In addition, two meals per day were provided in the latter two studies while hourly meal feeding was used in the present study. Our results are in line with Rutherfurd *et al.*^(40)^ who reported that apparent Ca absorption was minor in the stomach, substantial in the jejunum and negligible in the ileum. The negligible apparent Ca absorption in the distal-SI was probably caused by the low-Ca solubility under relatively high pH conditions. The reduction of apparent Ca digestibility from proximal to distal-SI in the absence of microbial phytase might indicate higher intestinal Ca secretion and/or reduction in the reabsorption of endogenous Ca due to chelation with dietary phytate. This assumption is supported by Lee *et al.*^(41)^ who reported that microbial phytase reduced basal endogenous Ca losses in growing pigs fed Ca-free diets. These studies do not allow to distinguish between a reduced secretion and enhanced reabsorption of endogenous secreted Ca in the later GIT segments.

Substantial Ca absorption was observed in the colon particularly in pigs fed phytase-free diets in the present study. The mechanism behind this effect might be that in phytase-free diets colonic microbiota degraded IP more than in phytase-supplemented diets, with subsequent higher release and absorption of IP bound Ca. Alternatively, although unlikely, the data might be influenced by segregation between the marker (Ti) and the nutrients of interest (see below). Our results conflict with Rutherfurd *et al.*^(40)^ who observed a similar apparent Ca digestibility in the ileum and faeces. Gonzalez-Vega *et al.*^(39)^ reported that standadised digestibility of Ca was significantly higher in the faeces than in the ileum of pigs fed *L. calcareum* but was similar between these two measurements in pigs fed Ca carbonate. These results indicate that colonic Ca absorption can be substantial depending on dietary Ca sources. Further studies, therefore, are warrented to clarify the mechanism of Ca absorption in the LI.

P was primarily absorbed in the SI and partly in the distal-LI. Microbial phytase might be most active in the stomach; thus, the P absorption site seemed to be affected by microbial phytase inclusion. A negative P absorption, that is, P secretion, was observed between the distal-SI and the proximal-LI, with subsequent P absorption in the distal-LI (Table 4). Colonic P secretion is supported by data reported by Larsen and Sandstrom^(42)^ who demonstrated a higher ileal compared with faecal apparent P digestibility in cannulated pigs. Our findings conflicted with Mesina *et al.*^(38)^ and Rosenfelder-Kuon *et al.*^(35)^ who demonstrated a similar ileal and faecal apparent P digestibility. Nonetheless, none of these studies including the present can exclude secretion and reabsorption of P in the caecum and colon. Our results are in line with Gonzalez-Vega *et al.*^(39)^ who reported that colonic P absorption was minor in IP free but substantial in IP enriched semi-purified diets. IP were hardly detected in the faeces of pigs even when remarkable amounts of IP entered the LI, indicating intense microbial IP degradation in the LI ^(35; 43)^. Colonic microbiota also in the present study may have degraded IP and released IP bound Ca and P more for the phytase-free diets; hence, a higher colonic P absorption was observed. Existence of colonic P absorption was proven by Liu *et al.*^(44,45)^, who reported that P absorption in the colon was substantial (10%) from diets containing maize distiller’s dried grains with solubles, while in diets containing soyabean meal and rapeseed meal, colonic P absorption was less (3–4%). They concluded that colonic P absorption was not affected by dietary P content but was highly dependent on dietary P sources. The colon, therefore, can have a significant capacity to absorb P depending on the diet. Mechanisms for colonic P absorption in pigs, however, remain largely unknown, that is, whether it is mediated via active transcellular routes or paracellular permeation. Another possible explanation for the fluctuation of apparent P digestibility in the colon might be that Ti transiently segregated from the digesta, which was observed in the gizzard and caeca in our previous broiler study^(46)^. However, this assumption is less likely under steady-state conditions with a constant passage rate of digesta in the GIT. Moreover, apparent DM digestibility gradually increased along the GIT as expected (online Supplementary Table S2), hence the marker (Ti) appeared to function well. A possible exception may have occurred in the stomach where a negative apparent digestibility of DM and Ca was observed, suggesting that TiO_2_ might have segregated from the digesta. Alternatively, this observation can also be ascribed to endogenous gastric Ca secretion. A negative apparent digestibility of Ca in the stomach was also observed by Rutherfurd *et al.*^(40)^.

### Calcium and phosphorus retention and excretion

An adequate dietary Ca/P is essential for P deposition in bone. Dietary phytase inclusion enhanced both absorption and retention of Ca and P, with a larger effect in medium and high Ca supplemented diets. In the low-Ca diets, the Ca supply limited P retention as indicated by the relatively high urinary P excretion. Despite a reduction in ATTD of P, the increase in dietary Ca from 2·0 to 5·8 g/kg increased the P retention, rP/dP, bone ash content and bone mineral mass, in particular in the phytase-supplemented diets as indicated by the interaction. The high retention of both ingested P (94%) and Ca (98%) at this treatment indicates that the digestible Ca to P ratio was close to optimum in this treatment. The highest retention of ingested P was realised at the medium dietary Ca content. The increase from medium to the highest Ca content reduced the P retention because of a reduction in ATTD of P. These results indicate that for optimal dietary P utilisation the effects of Ca on absorption and retention of P must be taken into account.

### Conclusions

A high dietary Ca content reduces small intestinal apparent P digestibility to a greater extent in phytase-supplemented diets via reducing IP degradation and precipitation of P. Dietary Ca is primarily absorbed in the proximal-SI and to a lesser extent in the LI in pigs, while P is mostly absorbed in the proximal and distal-SI with minor net absorption occurring in the LI. An adequate dietary Ca/P is essential for optimal P absorption and post-absorptive utilisation.

## Supplementary material

For supplementary material accompanying this paper visit https://doi.org/10.1017/S0007114522001039. click here to view supplementary material
