# Supplementary material for: The impact of dietary calcium content on phosphorus absorption and retention in growing pigs is enhanced by dietary microbial phytase supplementation
Source: Br J Nutr. 2022 Apr 13;129(6):955–66. doi: 10.1017/S0007114522001039 (PMC9991841; doi:10.1017/S0007114522001039)
Supplement: Supplementary file 2 [file S0007114522001039sup.zip › S0007114522001039sup002.odt]

| Supplementary table 1. Mean digesta pH of growing pigs as affected by dietary calcium (Ca) content and microbial phytase supplementation1,2 |  |
| --- | --- |
| Ca content g/kg | Phytase content FTU/kg | Stomach |  | Small intestine |  | Caeca |  | Large intestine |
| Proximal | Distal | Proximal | Distal |
| 2.0 | 0 | 3.2 |  | 5.8 | 6.6a |  | 5.5 |  | 5.4 | 5.8 |
| 5.8 | 0 | 4.1 |  | 6.0 | 6.2bc |  | 5.5 |  | 5.5 | 5.9 |
| 9.6 | 0 | 4.1 |  | 5.7 | 6.2c |  | 5.8 |  | 5.7 | 6.0 |
| 2.0 | 500 | 3.0 |  | 6.0 | 6.5a |  | 5.4 |  | 5.4 | 5.8 |
| 5.8 | 500 | 3.5 |  | 6.0 | 6.5ab |  | 5.4 |  | 5.5 | 6.0 |
| 9.6 | 500 | 4.0 |  | 5.8 | 6.6a |  | 5.6 |  | 5.7 | 6.1 |
| SEM | 0.21 |  | 0.11 | 0.11 |  | 0.09 |  | 0.07 | 0.10 |  |
| P-value |  |  |  |  |  |  |  |  |  |  |
| Ca content | <0.001 |  | 0.020 | 0.099 |  | 0.003 |  | <0.001 | 0.051 |  |
| Phytase content | 0.005 |  | 0.081 | 0.010 |  | 0.059 |  | 0.839 | 0.256 |  |
| Ca content × phytase content | 0.262 |  | 0.193 | 0.022 |  | 0.824 |  | 0.698 | 0.806 |  |
| a-c Values without common superscript within a column differ significantly (P≤0.05). 1 Dietary P content was fixed at 4.7 g/kg of diet, hence the Ca/P ratio was 0.4, 1.2 and 2.0 for the three increasing dietary Ca contents. 2 Values are presented as least square means and pooled standard errors of the mean, n 10. |  |

| Supplementary table 2. Mean apparent digestibility (%) of dry matter in different gastrointestinal tract of growing pigs as affected by dietary calcium (Ca) content and microbial phytase supplementation1,2 |
| --- |
| Ca content g/kg | Phytase content  FTU/kg | Stomach |  | Small intestine |  | Large intestine |
| Proximal | Distal | Proximal | Distal |
| 2.0 | 0 | -75.2 |  | 27.1 | 59.7 |  | 76.4 | 78.4 |
| 5.8 | 0 | -41.0 |  | 35.8 | 59.4 |  | 75.2 | 77.6 |
| 9.6 | 0 | -47.7 |  | 21.4 | 55.9 |  | 77.5 | 78.0 |
| 2.0 | 500 | -81.3 |  | 40.4 | 65.6 |  | 75.8 | 79.7 |
| 5.8 | 500 | -64.2 |  | 38.5 | 61.3 |  | 77.8 | 78.8 |
| 9.6 | 500 | -52.0 |  | 26.5 | 58.3 |  | 76.2 | 79.1 |
| SEM | 16.5 |  | 7.43 | 2.47 |  | 1.35 | 0.94 |
| P-value |  |  |  |  |  |  |  |
| Ca content | 0.041 |  | 0.050 | 0.011 |  | 0.716 | 0.406 |
| Phytase content | 0.213 |  | 0.123 | 0.034 |  | 0.784 | 0.035 |
| Ca content × phytase content | 0.603 |  | 0.549 | 0.379 |  | 0.146 | 0.988 |
| 1 Dietary P content was fixed at 4.7 g/kg of diet, hence the Ca/P ratio was 0.4, 1.2 and 2.0 for the three increasing dietary Ca contents. 2 Values are presented as least square means and pooled standard errors of the mean, n 10. |
